# Combination of PCT, sNFI and dCHC for the diagnosis of ascites infection in cirrhotic patients

**DOI:** 10.1186/s12879-018-3308-1

**Published:** 2018-08-10

**Authors:** Han Wang, Yan Li, Fangfang Zhang, Ning Yang, Na Xie, Yuanli Mao, Boan Li

**Affiliations:** 1 Clinical Diagnostic Centre, 302 Military Hospital of China, Beijing, 100039 People’s Republic of China; 2 Out-patient department, 302 Military Hospital of China, Beijing, 100039 People’s Republic of China

**Keywords:** Cirrhosis, Ascitic fluid, Procalcitonin, dCHC, sNFI

## Abstract

**Background:**

It is difficult to diagnose ascites infection early in cirrhotic patients. The present study was to create and evaluate a new bioscore combined with PCT, sNFI and dCHC in the diagnosis of ascites infection in cirrhotic patients.

**Methods:**

Two hundred and fifty-nine consecutive patients were enrolled; of which 51 patients were culture-positive spontaneous bacterial peritonitis (culture-positive SBP) and 58 patients were culture-negative SBP. The efficacy of procalcitonin(PCT), c-reactive protein (CRP), white blood cell (WBC), mean fluorescence intensity of mature neutrophils(sNFI) and difference in hemoglobin concentration between newly formed and mature red blood cells(dCHC) for diagnosing ascites infection was examined. These parameters were used to create a scoring system. The scoring system was analyzed by logistic regression analysis to determine which parameters were statistically different between ascites infection and non-ascites infection patients. Receiver operating characteristic curve (ROC) was used to analyze the diagnostic ability of bioscore for ascites infection.

**Results:**

In ROC analysis, the area under the curves (AUC) for PCT was 0.852 (95% CI 0.803–0.921, *P* < 0.001), dCHC 0.837 (95% CI 0.773–0.923, *P* < 0.001), CRP 0.669 (95% CI 0.610–0.732, *P* = 0.0624), sNFI 0.838 (95% CI 0.777–0.903, *P* < 0.001), and WBC 0.624 (95% CI 0.500–0.722, *P* = 0.0881). Multivariate analysis revealed PCT, dCHC and sNFI to be statistically significant. The combination of these three parameters in the bioscore had an AUC of 0.937 (95% CI 0.901–0.994, *P* < 0.001). A bioscore of ≥3.40 was considered to be statistically significant in making a positive diagnosis of ascites infection. In different groups of ascites infection, bioscore also shown a high diagnostic value of AUC was 0.947(95% CI 0.882–0.988, *P* < 0.001) and 0.929 (95% CI 0.869–0.974, *P* < 0.001) for culture-positive SBP and culture-negative SBP group respectively.

**Conclusion:**

The composite markers of combining PCT, dCHC and sNFI could be a valuable diagnostic score to early diagnose ascites infection in patients with cirrhosis.

**Electronic supplementary material:**

The online version of this article (10.1186/s12879-018-3308-1) contains supplementary material, which is available to authorized users.

## Background

Ascites infections are considered intrications in cirrhotic patients with increasing mobility and mortality [1]. The initial diagnosis of ascites infection is primarily to enhance patient survival [2]. Thus ascitic puncture is performed compulsorily for patient with ascites, whether suspected of infection or not [3].

The diagnosis of ascites infections cases are based on proof of the independent number of polymorphonuclear cells(PMN) in the infected fluid ≥0.25 × 10^9^cells/L, which is the most accurate sensitive value [4]. In sequence not to miss a case of ascitic fluid infection, hemorrhagic case of ascites or bacterascites, the PMN is not accurate to be used as an indicator of infection [2, 5]. The negative reading of the ascites culture is been as 60% of patients with suspicion of ascites infection [6]. Furthermore, the time and availability of ascites are not constant at all-time [7], therefore, is essential to develop sensitive, accurate and rapid methods to diagnose ascitic fluid infection.

Efforts have been made to develop new biomarkers that accurately diagnose bacterial infection. The serum PCT concentrations increase in patients with bacterial infections or sepsis [8], and is not elevated in viral or autoimmune diseases of the liver [9]. sNFI and dCHC is based on early immune reactions in systemic infection, has also displayed promise in discriminating between patients with and without infection [10–12]. As we known, neutrophil granulocyte is the most common immune cells in the first line of pathogen defense. And monocyte/macrophage involved in inflammation-induced anemia result in an immediate decrease in hemoglobin synthesis. sNFI and dCHC were easily measured from whole blood samples on a blood cell analyzer. sNFI represented mean fluorescence intensity of mature neutrophils. Blood cell analyzer measured sNFI in a fluorescence flow channel by determining the maximum fluorescence-peak-height from mature neutrophils. There is no influence from non-segmented neutrophils. sNFI represents neutrophil granulocyte activation. dCHC was difference in cellular hemoglobin content of young erythrocytes, freshly release from bone marrow, and, mature, peripherally circulating erythrocytes. dCHC represents the difference in hemoglobin concentration of newly formed red blood cells compared to mature ones. Accordingly, dCHC indirectly determines monocyte/macrophage activation [12]. An important advantage of sNFI and dCHC is that they require only the machine used for white blood cell count (WBC) counting [13].

Hematocytopenia is a serious complication in patients with cirrhosis, mainly manifesting as a multi-hemocyte decrease and only rarely as a decrease in one cell type [14, 15]. It raises doubts about whether sNFI and dCHC derived from blood cells could be a reliable marker for ascites infection diagnosis. Moreover, hematocytopenia in patients with cirrhosis varies in form and degree, and the previously determined cutoff values of sNFI and dCHC in discriminating between patients with and without infection may not be applicable in the presence of cirrhosis.

As well as we known, systemic inflammatory response to ascites infection is complex, a diagnostic score derived from a combination of different parameters would be more accurate in diagnosing ascites infection. The aim of this prospective study was to evaluate the value of these individual markers including WBC, PCT, CRP, dCHC and sNFI, and to establish a bioscore for increasing sensitivity and specificity on accurately early diagnosis of ascites infection. These biomarkers are all tested in blood, which is the main advantage of measuring these biomarkers over simply measuring WBC in ascitic fluid.

## Methods

### Study design and patients

This prospective study performed in four 48-bed Medical Departments in the 302 Military Hospital of China, Beijing, China, between December 2016 and July 2017. The study was approved by the Ethics Committee of the 302 Military Hospital of China (Beijing; Permit Number 2017–114). All patients or their parent or legal guardian provided written informed consent prior to study inclusion, at the time of submission.

Patients were included if 1) complete clinical information on complications and liver function assessments were available, and 2) all tests (pathogen cultures, WBC, CRP, PCT, dCHC, sNFI, blood and ascitic fluid biochemistry) had been conducted before antibiotic treatment. Patients were excluded if they had severe brain, heart, lung, psychiatric disease; serious fungal infection; tuberculosis; hepatocellular carcinoma; or secondary peritonitis. Severe brain disease included the categories of infections, trauma, stroke, seizures, tumors and also traumatic brain injury brain diseases [16]. Severe heart disease was defined as congenital cardiovascular defects, cardiomyopathy and heart failure, rheumatic heart disease, bacterial endocarditis, coronary heart disease and acute coronary syndrome [17]. Severe lung disease was defined as lung cancer, pneumonia, pulmonary fibrosis, asthma and chronic obstructive pulmonary disease [18]. The diagnosis of hepatic encephalopathy was according to the West Haven Classification [19].

### Paracentesis and culture techniques

All patients underwent diagnostic paracentesis and ascitic fluid culture. Ascitic fluid was drawn and collected in ethylenediaminetetraacetic acid tubes for analysis of leukocyte counts and biochemistry. A smear was carried out and stained with Gram stain. Peritoneal fluid collected from patients was cultured via two methods. Initially, ascitic fluid was collected into an aerobic blood culture bottle (10 mL) and an anaerobic blood culture bottle (10 mL) (bioMérieux; Durham, NC, USA), which were then incubated in an automated BacT/ALERT 3D (bioMérieux) culture system for 7 days. The conventional culture methods were also conducted, including inoculation of conventional agar and broth media at 35 °C for up to 72 h before being discarded as negative [3, 4, 20].

Culture parameters associated with higher contamination rates included microbial growth from a single specimen, isolation of certain skin flora and microbial species (eg, coagulase-negative Staphylococcus, Bacillus species and Diphtheroids), and longer time to detect growth in culture [21, 22]. Growth of a known skin flora in a solitary culture indicated contaminant. Growth of a known skin flora in more than one bottle in a multiple culture indicated pathogen [21].

### Classification of ascitic fluid infection

Ascitic fluid infection was determined and classified according to the method described previously [3, 4]. Briefly, ascitic fluid PMNL > 0.25 × 10^9^cells/L with positive bacterial culture indicated culture-positive SBP; PMNL > 0.25 × 10^9^cells/L, with negative Gram stain and bacterial culture indicated culture-negative SBP; and PMNL < 0.25 × 10^9^cells/L with positive bacterial culture indicated bacterascites. Sterile ascites was defined as PMNL < 0.25 × 10^9^cells/L with negative bacterial culture.

### Assessments

Every patient was assessed daily for features of ascitic fluid infection; when infection was suspected, samples were collected for bacteriological culture. Blood samples were obtained from an arterial line upon admission and subsequently daily at 07:00. Day 0 (D_0_) was the day of diagnosis of ascitic fluid infection (for the culture-positive SBP group) or the day of first suspicion of infection or the day of first ascitic fluid culture (for culture-negative SBP and sterile ascites groups). The criteria for a suspected infection was defined as one of the following criteria, as revised from available guidelines: abdominal pain and/or fever (Temperature > 37.5 °C), and/or abdominal pain and rebound tenderness [6, 23, 24].

The efficacy of PCT, CRP, WBC, sNFI and dCHC for diagnosing ascites infection was examined. These parameters were used to create a scoring system. To obtain the optimum sensitivity and specificity for detecting ascites infection, we developed the bioscore, the measured variable using an integer corresponding to its classification value (Additional file 1: Table S3). The scoring system was analyzed by logistic regression analysis to determine which variables were statistically different between ascites infection and non-ascites infection patients. The biomarkers which were not found diagnostic value in recognizing ascitic fluid infection were deleted from the scoring system. And finally, the total bioscore of each patient was established though two steps as showed in Additional file 2: Table S5.

WBC, sNFI, dCHC, PCT and CRP are measured in blood. WBC, sNFI and dCHC blood specimens were collected in K_3_EDTA tubes (Sarstedt, Germany). Blood for PCT and CRP measurement was sampled in a z serum clot activator tube. Samples were measured promptly after collection but within a maximum of 2 h without predilution. The coefficient of variation (CV) of WBC, dCHC, sNFI, PCT and CRP were calculated [25]. Reference values of sNFI and dCHC in healthy individuals were: sNFI: 420 ± 19.3FI-ch and dCHC: 0.4–7.0 pg.

WBC, sNFI, dCHC and ascitic fluid samples were quantified by fluorescence flow cytometry (XE-5000; Sysmex, Kobe, Japan). PCT level in serum was measured by the electrochemiluminescence immunoassay method (Roche Diagnostics, Mannheim, Germany) with a detection limit of 0.02 ng/mL using an immunology analyzer (Cobas E601; Roche Diagnostics, Mannheim, Germany). Total bilirubin, alanine aminotransferase, aspartate aminotransferase, albumin, CRP, glucose, and lactate dehydrogenase level in serum or ascitic fluid were measured using an AU680 analyzer (Beckman Coulter, Fullerton, USA).

### Statistical analysis

Data were summarized as mean ± standard deviation (SD) or percentages. Sample distribution was assessed by the Kolmogorov-Smirnov test. The Mann-Whitney or Kruskall-Wallis tests were used for comparison of continuous variables between groups, and the chi-square test or Fisher’s exact test were used for comparison of categorical variables. AUCs (with 95% confidence intervals) were calculated to assess the diagnostic values of the tests; AUCs > 0.70 were considered clinically relevant [26]. Logistic regression was to determine which parameters were statistically different between ascites infection and non-ascites infection patients. The bioscore system was analyzed for AUC, and the subsequent ROC curves were used to evaluate the prognostic value. The Youden index was applied to set the cutoffs and compared between the combined evaluation method and single evaluation methods. Statistical significance was set at two-tailed *P* ≤ 0.05. SAS, version 9.3 (SAS Institute Inc., Cary, NC, USA) was used for data analysis.

## Results

### Baseline population characteristics

**Fig. 1:**
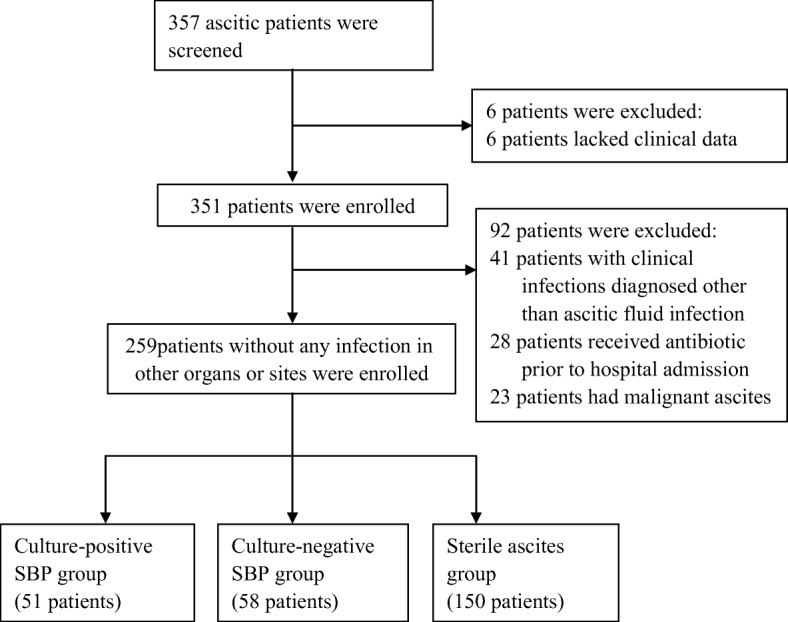
Study algorithm. SBP: spontaneous bacterial peritonitis

From among 357 consecutive ascites patients treated during the study period, 98 patients were excluded: 6 patients lacked clinical data were excluded; 41 patients were excluded because they had infections other than ascitic fluid infection; 28 because they had received antibiotics prior to hospital admission or enrollment in the study; and 23 because they had malignant ascites (Fig. 1). Finally, a total of 259 patients (mean age, 51.4 ± 19.7 years; 62.5% males) were included in the study; of these, 51/259 (19.7%) had culture-positive SBP, 58/259 (22.4%) had culture-negative SBP, and 150/259 (57.9%) had sterile ascites (Fig. 1). According to etiology, hepatitis B cirrhosis was the most common (84/259; 32.4%). Child-Pugh stage C disease was present in 153/259 (59.1%) patients (Additional file 3: Table S1).

### Ascitic fluid analysis

The analysis of ascitic fluid showed WBC count of 4.8 (1.3) × 10^9^cells/L, 3.3(1.1) × 10^9^cells/L, and 0.2(0.06) × 10^9^cells/L in culture-positive SBP, culture-negative SBP and sterile ascites, respectively (Additional file 3: Table S1). Culture was positive in 51/259 (19.7%) patients. Gram-negative infection was the most common (26/51; 51.0%), and *Escherichia coli* the most common organism (15/51; 29.4%); the other causative organisms were *Staphylococcus epidermidis* (11/51; 21.6%), *Klebsiella pneumonia* (7/51; 13.7%), *Enterococcus faecium* (6/51; 11.8%), *Staphylococcus haemolyticus* (4/51, 7.8%), *Pseudomonas aeruginosa* (3/51; 5.9%), *Staphylococcus hominis* (2/51; 3.9%), *Micrococcus luteus* (2/51; 3.9%), and *Acinetobacter baumannii* (1/51. 2.0%).

### The diagnostic accuracy of the bioscore for ascitic fluid infection

**Fig. 2:**
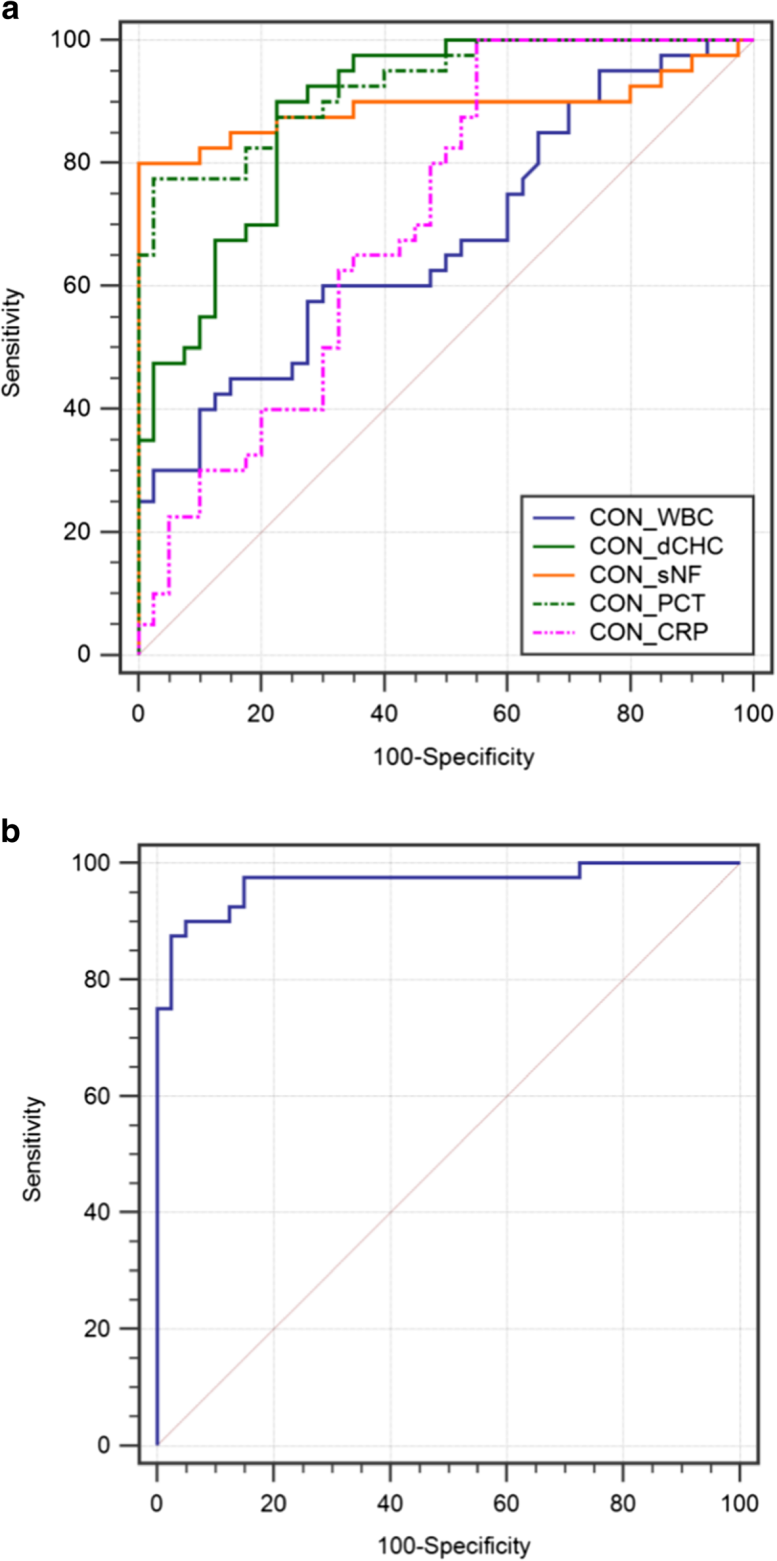
ROC curve of clinical markers for diagnosis of ascitic fluid infection. **a** AUC for PCT was 0.852 (95% CI 0.803–0.921, *P* < 0.001), dCHC 0.837 (95% CI 0.773–0.923, *P* < 0.001), CRP 0.669 (95% CI 0.610–0.732, *P* = 0.0624), sNFI 0.838 (95% CI 0.777–0.903, *P* < 0.001), and WBC 0.624 (95% CI 0.500–0.722, *P* = 0.0881). For calculated score: AUC for a null hypothesis was 0.5. **b** AUC for calculated score was 0.937 (95% CI 0.901–0.994, *P* < 0.001). For calculated score: AUC for a null hypothesis was 0.5. ROC: receiver operating curve, AUC: area under the curve, CON: for all patients

In ROC analysis, the AUCs of PCT, dCHC, CRP, sNFI and WBC were 0.852, 0.837, 0.669, 0.838 and 0.624, respectively (Additional file 4: Table S2). PCT, dCHC and sNFI were significant for the diagnosis of ascitic fluid infection in patients with cirrhosis (Fig. 2a). The optimal cut-off values were 0.88 ng/mL, 0.56 pg, 15.4 mg/L, 550FI-ch, and 8.7 × 10^9^/L. The CV values of WBC, dCHC, sNFI, PCT and CRP were 2.5%, 2.6%, 2.1%, 2.0% and 3.1% respectively.

To facilitate the development of an accurate bioscore to ascitic fluid infection diagnosis, we enstablished a categorical bioscore to better describe the syndrome. To obtain the optimum sensitivity and specificity for detecting ascites infection, we developed the bioscore, the measured variable using an integer corresponding to its classification value (Additional file 1: Table S3). Three multiple logistic regression model were constructed for culture-positive SBP, culture-negative SBP and all patients group respectively. Each of the five markers was a separate model to evaluate its performance in the diagnosing culture-positive SBP, culture-negative SBP and all patients group respectively. The variables determined by the analysis that showed in Additional file 5: Table S4 found statistical significance of PCT, dCHC and sNFI in both three groups. WBC and CRP had no diagnostic value in diagnosing culture-positive SBP, culture-negative SBP and combined group. Accordingly, WBC and CRP were eliminated from total bioscore. We analyzed each patient by PCT, dCHC and sNFI for the total bioscore using a ROC curve.

The AUC of the bioscore was 0.937 (95% CI 0.881–0.984, *P* < 0.001) for the diagnosis of ascitic fluid infection (Fig. 2b), at the cutoff value of 3.40. Meanwhile, the sensitivity, specificity, positive likelihood ratio and negative likelihood ratio of the bioscore were 92.6%, 95.3%, 18.6 and 0.11, respectively (Additional file 4: Table S2).

### The diagnostic accuracy of the bioscore in subgroup populations of ascitic fluid infection

**Fig. 3:**
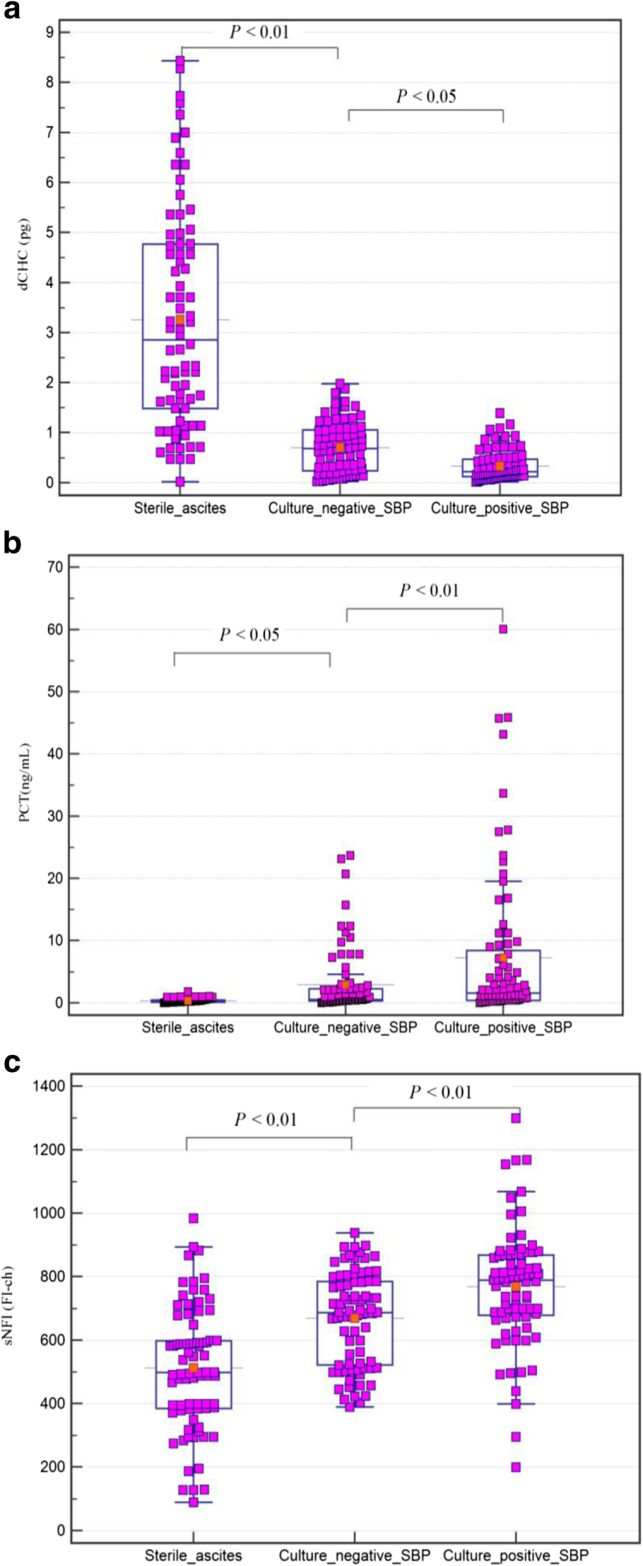
Comparison of dCHC(**a**), PCT(**b**) and sNFI(**c**) according to the ascitic fluid infection groups. In the box-and-whisker plot, the central box represents the interquartile range (IQR); the middle line represents the median

In the current study, the values of dCHC, PCT and sNFI in culture-positive SBP patients were significantly higher than in culture-negative SBP patients (0.3 pg vs. 0.7 pg, *P* < 0.05; 7.2 ng/mL vs.2.9 ng/mL, *P* < 0.01; 804.6FI-ch vs. 653.6FI-ch, *P* < 0.01, respectively) (Fig. 3). Therefore, from sterile ascites, through culture-negative SBP to culture-positive SBP, the variation of dCHC and sNFI showed an increasing trend. Culture-positive SBP and culture-negative SBP have a similar clinical course, but the complications are more frequent in the former. Different mechanisms of ascitic infection procedures impact physiologic parameters. Therefore, the diagnostic ability of the bioscore in different ascites infection subgroups should be assessed.

**Fig. 4:**
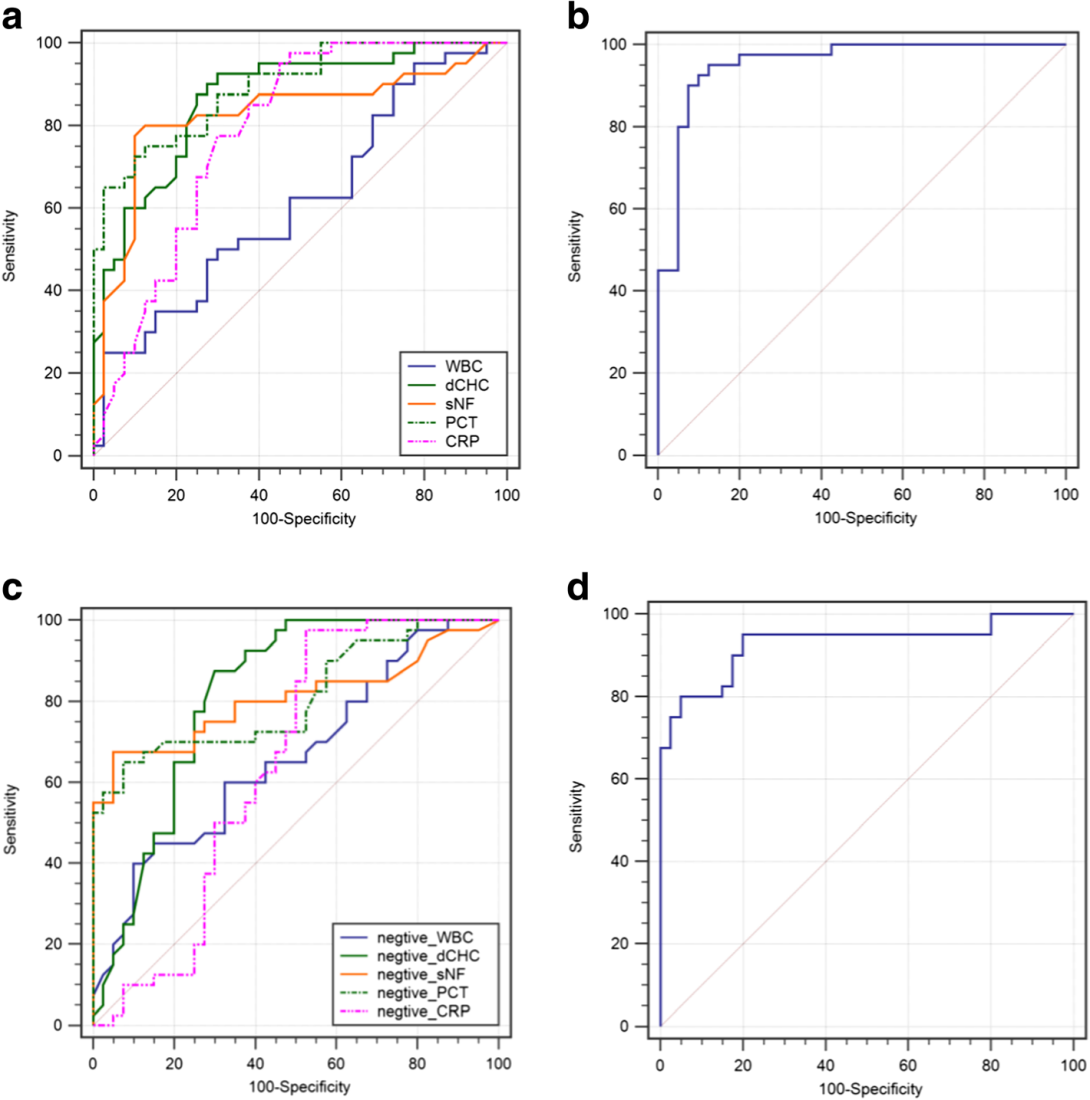
ROC curve of clinical markers for diagnosis of ascitic fluid infection. **a** ROC curve of clinical markers for diagnosis of culture-positive SBP. AUC for PCT was 0.865 (95% CI 0.814–0.932, *P* < 0.001), dCHC 0.849 (95% CI 0.799–0.918, *P* < 0.001), CRP 0.676 (95% CI 0.597–0.746, *P* = 0.0561), sNFI 0.857 (95% CI 0.802–0.920, *P* < 0.001), and WBC 0.627 (95% CI 0.563–0.701, *P* = 0.0783). For calculated score: AUC for a null hypothesis was 0.5. **b** ROC curve of calculated score for diagnosis of culture-positive SBP. AUC was 0.947 (95% CI 0.882–0.988, *P* < 0.001). AUC for a null hypothesis was 0.5. **c** ROC curve of clinical markers for diagnosis of culture-negative SBP. AUC for PCT was 0.809 (95% CI 0.726–0.888, *P* < 0.001), dCHC 0.816 (95% CI 0.753–0.874, *P* < 0.001), CRP 0.645 (95% CI 0.530–0.739, *P* = 0.0667), sNFI 0.807 (95% CI 0.737–0.887, *P* < 0.001), and WBC 0.615 (95% CI 0.550–0.686, *P* = 0.0774). For calculated score: AUC for a null hypothesis was 0.5. **d** ROC curve of calculated score for diagnosis of culture-negative SBP. AUC for calculated score was 0.929 (95% CI 0.869–0.974, *P* < 0.001). AUC for a null hypothesis was 0.5. ROC: receiver operating curve, AUC: area under the curve

In the group of culture-positive SBP (*n* = 51), PCT, dCHC and sNFI were significant for the diagnosis of ascitic fluid infection (Fig. 4a; Additional file 4: Table S2). The AUCs were 0.865, 0.849 and 0.857 for PCT, dCHC and sNFI, respectively. For the established bioscore, the AUC was 0.947 (95% CI 0.882–0.988, *P* < 0.001), while the sensitivity and specificity were 92.5% and 97.4%, respectively. The diagnostic accuracy of bioscore was higher than that of the any individual makers (Fig. 4b; Additional file 4: Table S2).

In the group of culture-negative SBP (*n* = 58), PCT, dCHC and sNFI were also significant for the diagnosis of ascitic infection (Fig. 4c; Additional file 4: Table S2). For the bioscore, the AUC was 0.929 (95% CI 0.869–0.974, *P* < 0.001), which was also higher than that of the any individual makers in the diagnosis of ascitic fluid infection (Fig. 4d; Additional file 4: Table S2).

## Discussion

Rapid detection of bacteria in the ascitic fluid and early diagnosis of ascitic infection is the key to improve the survival of cirrhotic patients with ascitic fluid [1].

The current study investigated the diagnostic ability of PCT, CRP, dCHC, sNFI and WBC, and showed that when threshold values of PCT, dCHC and sNFI were taken into consideration by calculation of a bioscore value, this could be considered a statistically significant diagnostic score for ascitic fluid. The bioscore demonstrated impressive accuracy for early diagnosis of ascitic fluid infection. These biomarkers are all tested in blood. Compared to simply measuring WBC in ascitic fluid, the biomarkers will provide a new and feasible choice for clinical practice.

In the present study, 42.1% of patients with cirrhosis had ascitic fluid infection, without infection elsewhere; this prevalence is comparable with previous reports [4] [27]. It is noted that no patient had bacterascites in the current study. Actually, we believed that the study of biomarkers for diagnostic accuracy of bacterascites was more complex than that of culture-positive SBP and culture-negative SBP, because it was still controversial whether bacterascites require a prompt initiation of antibiotherapy [3, 28]. The clinical diagnosis and antibiotherapy of bacterascites may divide into different groups according to the further ascites neutrophils count and ascites culture. And in order to get more powerful judgments, we suggested that the study of biomarkers for diagnostic accuracy of bacterascites needs further results based on clinical diagnosis and also a large number of cases.

Culture was positive in 51/259 (19.7%) of the sample; this is lower than the incidences previous reports [29–32]. *E. coli*, *K. pneumoniae*, and other Enterobacteriaceae are most likely to cause SBP by bacterial translocation [3]. Diverse organisms were isolated in our culture positive patients but, consistent with earlier reports, the most frequently isolated were *E. coli* (29.4%), *S. epidermidis* (21.6%), and *K. pneumonia* (13.7%).

Various biomarkers have been tested for their potential for use in rapid screening tests for infection [33–37]. The discriminative capabilities of PCT and CRP for ascites infection in patients with cirrhosis in this study are in line with but a little lower than previous reports, where the AUCs of PCT have ranged from 0.89 to 0.94 (sensitivity 30–95%; specificity 70–98%) and that of CRP from 0.75 to 0.78 (sensitivity 64–75%; specificity 61.2–95%) [24, 27, 38, 39]. This difference between the studies is mainly due to differences in the study population. The AUC of CRP was lower than that of PCT in all groups, which finding is also consistent with previous studies [4]. Additionally, previous research has suggested that while the basal level of CRP in cirrhotic patients is higher than in non cirrhotic patients, the degree of increase in CRP is less when liver function is impaired during infection. Thus, it appears that CRP is relatively less diagnostic value of infection in patients with advanced cirrhosis [40, 41]. Accordingly, it may explain why the significant lower AUCs for CRP (0.645–0.676) in the current study for diagnosis of ascitic fluid infection. WBC is a traditional marker for infection. Our data support the view of some authors that peripheral blood WBC has little value for diagnosing SBP [42].

Unlike WBC count, which relies on increased cells numbers to respond to infection, new biomarkers of sNFI directly reflects the inflammatory activity of both existing and increased WBC. dCHC involved in the early innate immune response and the reflected bone marrow production of innate immune cells. Hematologic indices (HI) were frequently abnormal in patients with cirrhosis especially with decompensated cirrhosis [43]. The baseline levels of sNFI and dCHC may have been affected by abnormal HI and varies in degree. However, a previous study found that only 3% of cirrhosis patients had abnormal bone marrow biopsy results [44]. And we speculated that in ascites infection, the levels of sNFI and dCHC in patients with cirrhosis were unaffected or only partially affected by cirrhosis. In the current study, both dCHC and sNFI showed good diagnostic performance in diagnosing ascitic fluid infection in patients with cirrhosis. The AUCs of dCHC and sNFI compared with that of PCT, 0.837 and 0.838, respectively. Of note, using the best cut-offs, dCHC showed high sensitivity /low specificity of 92.5%/70.0% and sNFI showed low sensitivity/high specificity of 77.5%/90.1%.

The complementary sensitivity/specificity profiles of each marker allowed the construction of a new bioscore which more discriminating and highly specific than each single component. Multivariate analysis found statistical significance of PCT, dCHC and sNFI, and the AUC of the total bioscore using those three markers was 0.937. The sensitivity and specificity of the bioscore were improved to 92.6% and 95.3%, respectively (Additional file 4: Table S2). The present study addressed that multi-marker approach may be an aid for the diagnosis of ascitic fluid infection.

It is known that complications are more frequent in culture-positive SBP than in culture-negative SBP and it may be involved in process and severity of ascites infection [45]. Therefore, the ability of biomarker for diagnosing ascitic fluid infection was studied separately in culture-positive and culture-negative groups. In the current study, 51 patients had culture-positive SPB and 58 had culture-negative SBP. The same as PCT [4, 45], we found a lower diagnostic value of dCHC, sNFI and bioscore in culture-negative SBP patients than those in culture-positive SBP patients. Our population was similar to our latest finding that dCHC and sNFI may associate with different mechanisms of ascitic infection.

Some relatively new biomarkers (lipopolysaccharide-binding protein, ascites leukocyte esterase activity, lactoferrin, and bacterial DNA) were useful for diagnosing infection [33–37]. Compared with them, PCT, dCHC and sNFI are available in most of the hospital laboratories, and their combination in an easily computable score could improve the accuracy of ascitic infection diagnosis in patients with cirrhosis. In each group of patients, including culture-positive SBP and culture-negative SBP group, this method provided a more reliable diagnostic score for ascitic infection patients with cirrhosis.

The bioscore could easily be incorporated into clinical practice, as the bioscore has the following advantages. Firstly, the biomarkers are all tested in blood. It means that compared with diagnostic paracentesis, haematological examination is less traumatic and patient compliance is better. Accordingly, haematological indicators analysis compared to ascitic fluid test is conducive to the routine monitoring of ascites infection. Secondly, the diagnostic value of this bioscore in ascites infection is higher than that of PCT and CRP, and these two commonly used ascites infection indicators. In addition, dCHC and sNFI can be available directly from the WBC detection. This means that the score does not increase any instrument compared with PCT. In particular, the bioscore is of great value for early diagnosis of ascites infection and avoiding unnecessary diagnostic paracentesis. If the bioscore of patient was showed positive, the patient is likely to have infection and requires paracentesis. The bioscore was especially clinical significant for the early diagnosis in culture-negative SBP group. Totally, 94.7% of cirrhotic patients with ascites can be directly identified as ascites infection by this score. More valuable is that, if negative by this bioscore, although 9.5% of cirrhotic patients with ascites used by the bioscore as false-negatives would have been observed, more than 90% of cirrhotic patients with ascites can exclude ascites infection, thus can be spared paracentesis for diagnosis. As a score that can be calculated only by PCT and WBC detection, it is very feasible for screening ascites infection that does not need diagnostic paracentesis. Furthermore, in the current study, the CV of each of those markers showed a lower value ranged between 2.0 and 2.6%, which mean that the assay has very small variability and high reproducibility.

This study has some limitations. This is a single-center study and should be considered as a pilot study defining the new bioscore and its cutoff values for diagnosis of ascites infection in patients with cirrhosis. As the development of a diagnostic scale is only the first step in research; the next steps would be external validation using the same cut-off points. Accordingly, another independent cohort of cirrhotic patients with ascites should validated the bioscore. Furthermore, external validation also should contain multicenter studies with larger samples to confirm our findings. As we know, different laboratories may have different study populations, ascitic fluid culture conditions and culture methods, even the transportation and storage of specimen. The validation of multicenter studies will bring the bioscore more power and make the results more generalizable.

## Conclusions

In conclusion, the present study suggests that the composite markers of combining PCT, dCHC and sNFI could be a valuable diagnostic score to early diagnose ascites infection in patients with cirrhosis.

## Additional files

Additional file 1 **Table S3.** The grading criteria of PCT, dCHC, sNFI, CRP and WBC. (DOC 37 kb)

Additional file 2: **Table S5.** The bioscore for ascitic fluid infection diagnosis. (DOC 36 kb)

Additional file 3: **Table S1.** Baseline demographic data and clinical variables of the enrolled patients. (DOC 55 kb)

Additional file 4: **Table S2.** Diagnostic accuracy of each marker in cirrhotic patients with ascitic fluid infection. (DOC 52 kb)

Additional file 5: **Table S4.** Multivariate associations of PCT, CRP, dCHC, sNFI and WBC with ascitic infection. (DOC 49 kb)

## Abbreviations

aIG#: Immature granulocyte count
ASL#: Antibody secreting lymphocyte count
AUCs: Area under the curve
CI: Confidence interval
CRP: C-reactive protein
dCHC: difference in hemoglobin concentration between newly formed and mature red blood cells
HAAA: Hepatitis-associated aplastic anemia
HVPG: Hepatic venous pressure gradient
ICIS: Intensive Care Infection Score
ICIS_Δ_: Adjusted ICIS
MDS RAEB-1: Myelodysplastic syndromes refractory anemia with excess of blasts-1
MDS RAEB-2: Myelodysplastic syndromes refractory anemia with excess of blasts-2
MELD: Model for end-stage liver disease
NPV: Negative predictive value
PCT: Procalcitonin;
PMNL: Polymorphonuclear leukocytes;
PPV: Positive predictive value
ROC: Receiver operating characteristic;
SBP: Spontaneous bacterial peritonitis;
sN#: Total segmented neutrophil count
sNFI: Mean fluorescence intensity of mature (segmented) neutrophils
WBC: White blood cell
