# Supplementary material for: Combination of PCT, sNFI and dCHC for the diagnosis of ascites infection in cirrhotic patients
Source: BMC Infect Dis. 2018 Aug 10;18:389. doi: 10.1186/s12879-018-3308-1 (PMC6086035; doi:10.1186/s12879-018-3308-1)
Supplement: Supplementary file 1 — Table S3. The grading criteria of PCT, dCHC, sNFI, CRP and WBC. (DOC 37 kb) [file 12879_2018_3308_MOESM1_ESM.doc]

| **Table S3** **The grading criteria of PCT, dCHC, sNFI, CRP and WBC.** | | |  |
| --- | --- | --- | --- |
| **Marker** | **Range** | **Scoring** |  |
| PCT (ng/ml) | <0.5 | 0 |  |
|  | 0.5 to 2 | 1 |  |
|  | 2 to 10 | 2 |  |
|  | >10 | 3 |  |
| dCHC (pg) | < 0.54 | 0 |  |
|  | 0.54 to 0.58 | 1 |  |
|  | > 0.58 | 2 |  |
|  |  |  |  |
| sNFI (FI-ch) | < 500 | 0 |  |
|  | 500 to 550 | 1 |  |
|  | > 580 | 2 |  |
|  |  |  |  |
| CRP (mg/L) | <8 | 0 |  |
|  | 8 to 50 | 1 |  |
|  | >50 | 2 |  |
| WBC (×10^9^/L) | 4 to 12 | 0 |  |
|  | <4 or >12 | 1 |  |
