# Supplementary material for: Combination of PCT, sNFI and dCHC for the diagnosis of ascites infection in cirrhotic patients
Source: BMC Infect Dis. 2018 Aug 10;18:389. doi: 10.1186/s12879-018-3308-1 (PMC6086035; doi:10.1186/s12879-018-3308-1)
Supplement: Supplementary file 2 — Table S5. The bioscore for ascitic fluid infection diagnosis. (DOC 36 kb) [file 12879_2018_3308_MOESM2_ESM.doc]

**Table S5 The bioscore for ascitic fluid infection diagnosis.**

Step 1.

| Score | PCT (ng/ml) |
| --- | --- |
| 0 | <0.5 |
| 1 | 0.5 to 2 |
| 2 | 2 to 10 |
| 3 | >10 |

| Score | dCHC (pg) |
| --- | --- |
| 0 | < 0.54 |
| 1 | 0.54 to 0.58 |
| 2 | > 0.58 |

| Score | sNFI (FI-ch) |
| --- | --- |
| 0 | < 500 |
| 1 | 500 to 550 |
| 2 | > 580 |

Step 2.

| Risk factor | Score |
| --- | --- |
| PCT | ________ |
| dCHC | ________ |
| sNFI | ________ |
| Total | ________ |
