# Supplementary material for: Combination of PCT, sNFI and dCHC for the diagnosis of ascites infection in cirrhotic patients
Source: BMC Infect Dis. 2018 Aug 10;18:389. doi: 10.1186/s12879-018-3308-1 (PMC6086035; doi:10.1186/s12879-018-3308-1)
Supplement: Supplementary file 3 — Table S1. Baseline demographic data and clinical variables of the enrolled patients. (DOC 55 kb) [file 12879_2018_3308_MOESM3_ESM.doc]

**Table S1** Baseline demographic data and clinical variables of the enrolled patients

| **Mean (SD) or number (%)** | **Total**  **(N = 259)** | **Culture-**  **positive SBP**  **(n = 51)** | **Culture-**  **negative**  **SBP**  **(n = 58)** | | **Sterile ascites**  **(n = 150)** | |  | | |
| --- | --- | --- | --- | --- | --- | --- | --- | --- | --- |
| **Physical examination** |  |  |  | |  | |  | | |
| Age (years) | 51.4 (19.7) | 54.5 18.3) | 48.6 (16.8) | | 52.7 (11.4) | |  | | |
| Sex (male) | 162 (62.5) | 26 (51.1) | 38 (65.5) | | 98 (65.3) | |  | | |
| **Etiology of liver cirrhosis**, **n (%)** | | | | | | |  | | |
| HBV-related cirrhosis | 84 (32.4) | 20 (39.2) | 22 (38.0) | | 42 (28.0) | |  | | |
| HCV-related cirrhosis | 72 (27.8) | 13 (25.5) | 11 (19.0) | | 48 (32.0) | |  | | |
| Alcoholic cirrhosis | 37 (14.3) | 8 (15.7) | 9 (15.5) | | 20(13.3) | |  | | |
| Autoimmune cirrhosis | 34(13.1) | 7 (13.7) | 9 (15.5) | | 18 (12.0) | |  | | |
| Cryptogenic cirrhosis | 32 (12.4) | 3 (5.9) | 7 (12.1) | | 22(14.7) | |  | | |
| **Child-Pugh class, n (%)** |  |  |  | |  | |  | | |
| Child A | 0 | 0 | 0 | | 0 | |  | | |
| Child B | 106 (40.9) | 13 (25.5) | 19 (32.8) | | 66 (44.0) | |  | | |
| Child C | 153 (59.1) | 38 (74.5) | 39 (67.2) | | 84(56.0) | |  | | |
| **MELD score, mean (±SD)** | 17.3 (7.5) | 18.6 (7.4) | 17.8 (8.3) | | 15.2 (7.5) | |  | | |
| **Complications, n (%)** |  |  |  | |  | |  | | |
| Hepatic encephalopathy | 80(30.8) | 39 (76.5) | 41 (70.7) | | 0 | |  | | |
| Hepatorenal syndrome | 39 (15.1) | 19 (37.3) | 20 (34.5) | | 0 | |  | | |
| Septic shock | 26 (10.0) | 13(25.4) | 13 (22.4) | | 0 | |  | | |
| **Laboratory tests D0, mean (SD)** | |  | |  |  | | |  | |
| WBC (×10^9^/L) | 7.9 (9.2) | 15.7 (8.0) | | 11.5 (8.9) | 4.1 (4.9) | | |  | |
| PMNL (×10^9^/L) | 6.2 (2.4) | 12.1 (6.9) | | 8.7 (4.7) | 2.2 (1.3) | | |  | |
| Lym (×10^9^/L) | 2.1 (2.1) | 2.5 (1.1) | | 2.0 (1.4) | 1.8 (0.8) | | |  | |
| RBC (×10^12^/L) | 3.6 (1.2) | 3.4 (0.9) | | 3.8 (1.1) | 3.7 (1.2) | | |  | |
| HGB (g/dL) | 95.6(22.4) | 89.3(18.8) | | 97.4 (29.2) | 101.7(35.2) | | |  | |
| ALB (g/L) | 30.6 (3.6) | 28.1 (2.8) | | 29.8 (3.7) | 31.8 (3.1) | | | |  |
| ALT (U/L) | 57.8 (35.6) | 65.7 (49.4) | | 55.4(35.8) | 51.6 (22.7) | | | |  |
| AST (U/L) | 68.9 (46.8) | 78.2 (40.7) | | 66.8 (32.7) | 61.4 (37.6) | | | |  |
| TBIL (μmol/L) | 64.6 (38.5) | 68.4 (41.7) | | 65.3 (33.3) | 57.4 (30.6) | | | |  |
| PCT(ng/mL) | 3.5 (8.5) | 7.2(9.8) | | 2.9(5.3) | 0.4(0.4) | | | |  |
| sNFII (FI-ch) | 656.4  (238.1) | 804.6 (232.6) | | 653.6(179.7) | 508.3 (201.6) | | | |  |
| dCHC (pg) | 1.4(1.8) | 0.3 (0.3) | | 0.7 (0.5) | 3.3 (2.2) | | | |  |
| CRP(mg/L) | 24.1(18.5) | 37.9(22.7) | | 32.6(19.6) | 7.3(15.2) | | | |  |
| **Ascitic fluid analysis D0, mean (±SD)** | | | | | | | | |  |
| WBC (×10^9^ cells/L) | 2.3 (1.7) | 4.8 (1.3) | | 3.3(1.1) | | 0.2(0.06) | | |  |
| PMNL (×10^9^ cells/L) | 2.1 (1.5) | 4.1 (1.6) | | 2.6 (1.3) | | 0.1 (0.07) | | |  |
| ALB (g/L) | 7.6 (2.7) | 8.1 (1.8) | | 8.0 (1.5) | | 7.2 (1.6) | | |  |
| Glu (mg/dL) | 109.1 (14.3) | 118.5 (17.3) | | 108.3 (11.9) | | 102.3 (10.8) | | |  |
| LDH (mU/mL) | 769.1 (389.7) | 2109.6  (358.7) | | 294.8  (87.6) | | 101.7  (37.6) | | |  |
| Protein (g/L) | 16.9 (3.3) | 16.6 (2.6) | | 16.8 (1.8) | | 17.3 (2.9) | | |  |
| **Length of hospital stay ( days), mean (±SD)** | 15.8 (11.7) | 17.7 (6.7) | | 15.6 (5.4) | | 12.8 (3.8) | | |  |
| **30-day mortality, n (%)** | 15.0 (4.9) | 11 (12.5) | | 4 (4.1) | | 0 | | |  |

SBP: Spontaneous bacterial peritonitis; MELD score: Model for End-Stage Liver Disease Score; HVPG: hepatic venous pressure gradient; WBC: white blood cell; PMNL: polymorphonuclear leukocyte count; Lym: Lymphocytes; RBC: red blood cell; HGB: hemoglobin; ALB: albumin; ALT: aminotransferase; AST: aspartate aminotransferase; TBIL: total bilirubin; PCT: procalcitonin; CRP: C-reactive protein; sNFII: mean fluorescence intensity of mature neutrophils; dCHC: difference in hemoglobin concentration between newly formed and mature red blood cells; Glu: glucose; LDH: lactate dehydrogenase.
