# Supplementary material for: Combination of PCT, sNFI and dCHC for the diagnosis of ascites infection in cirrhotic patients
Source: BMC Infect Dis. 2018 Aug 10;18:389. doi: 10.1186/s12879-018-3308-1 (PMC6086035; doi:10.1186/s12879-018-3308-1)
Supplement: Supplementary file 4 — Table S2. Diagnostic accuracy of each marker in cirrhotic patients with ascitic fluid infection. (DOC 52 kb) [file 12879_2018_3308_MOESM4_ESM.doc]

**Table S2 Diagnostic accuracy of each marker in cirrhotic patients with ascitic fluid infection**

| **Variables** | **AUC** | | **Cut-off value** | ***P* value** | **Sensitivity**  **(%)** | **Specificity**  **(%)** | **+LR** | **−LR** | **+PV** | **-PV** |
| --- | --- | --- | --- | --- | --- | --- | --- | --- | --- | --- |
| **All patients** | | |  |  |  |  |  |  |  |  |
| PCT | 0.852 | | 0.88 ng/mL | <0.0001 | 75.2 | 87.5 | 6.1 | 0.29 | 85.7 | 77.8 |
| dCHC | 0.837 | | 0.55 pg | <0.0001 | 92.5 | 70.0 | 3.1 | 0.11 | 75.5 | 90.3 |
| CRP | 0.669 | | 15.4 mg/L | 0.0505 | 89.5 | 45.3 | 1.6 | 0.28 | 61.4 | 78.3 |
| sNFI | 0.838 | | 550 FI-ch | <0.0001 | 77.5 | 90.1 | 7.8 | 0.25 | 88.6 | 80.8 |
| WBC | 0.624 | | 8.7×10^9^/L | 0.0581 | 40.3 | 90.9 | 4.3 | 0.67 | 80.7 | 60.4 |
| Score | 0.937 | | 3.40 | <0.0001 | 92.6 | 95.3 | 18.6 | 0.11 | 94.7 | 90.5 |
| **Culture-positive SBP** | | | |  |  |  |  |  |  |  |
| PCT | 0.865 | | 1.24 ng/mL | <0.0001 | 77.5 | 92.5 | 31.2 | 0.23 | 90.9 | 81.2 |
| dCHC | 0.849 | | 0.58pg | <0.0001 | 90.3 | 77.5 | 4.5 | 0.13 | 80.5 | 88.6 |
| CRP | 0.676 | | 39.6 mg/L | 0.0561 | 90.5 | 52.9 | 2.1 | 0.048 | 67.2 | 90.1 |
| sNFI | 0.857 | | 580 FI-ch | <0.0001 | 80.2 | 93.6 | 8.1 | 0.22 | 88.9 | 81.8 |
| WBC | 0.627 | | 12.8×10^9^/L | 0.0783 | 40.3 | 75.1 | 4.2 | 0.67 | 76.2 | 60.8 |
| Score | 0.947 | | 3.95 | <0.0001 | 92.5 | 97.4 | 9.3 | 0.083 | 93.2 | 92.3 |
| **Culture-negative SBP** | | | | | |  |  |  |  |  |
| PCT | | 0.809 | 0.41 ng/mL | <0.0001 | 65.6 | 92.5 | 8.6 | 0.38 | 89.7 | 72.5 |
| dCHC | | 0.816 | 0.54 pg | <0.0001 | 87.5 | 70.1 | 2.9 | 0.18 | 74.5 | 84.8 |
| CRP | | 0.645 | 27.2 mg/L | 0.0667 | 94.5 | 47.5 | 1.8 | 0.053 | 65.1 | 95.4 |
| sNFI | | 0.807 | 500 FI-ch | <0.0001 | 67.5 | 93.4 | 13.5 | 0.34 | 93.1 | 74.5 |
| WBC | | 0.615 | 8.1×10^9^/L | 0.0774 | 25.1 | 87.5 | 10.2 | 0.77 | 85.9 | 56.5 |
| Score | | 0.929 | 3.35 | <0.0001 | 92.1 | 93.3 | 4.75 | 0.063 | 89.6 | 94.1 |

AUC: area under receiver operating characteristics curve; +LR: positive likelihood ratio; **-**PV: negative likelihood ratio; +PV: positive predictive value; -PV: negative predictive value; PCT: procalcitonin; dCHC: difference in hemoglobin concentration between newly formed and mature red blood cells; CRP: C-reactive protein; sNFII: mean fluorescence intensity of mature (segmented) neutrophils; WBC: White blood cell.
