# Supplementary material for: Combination of PCT, sNFI and dCHC for the diagnosis of ascites infection in cirrhotic patients
Source: BMC Infect Dis. 2018 Aug 10;18:389. doi: 10.1186/s12879-018-3308-1 (PMC6086035; doi:10.1186/s12879-018-3308-1)
Supplement: Supplementary file 5 — Table S4. Multivariate associations of PCT, CRP, dCHC, sNFI and WBC with ascitic infection. (DOC 49 kb) [file 12879_2018_3308_MOESM5_ESM.doc]

**Table S4 Multivariate associations of PCT, CRP, dCHC, sNFI and WBC** with ascitic infection

| Marker | Culture-positive  SBP | |  | Culture-negative SBP |  |  | All patients |  |
| --- | --- | --- | --- | --- | --- | --- | --- | --- |
|  | OR (95%CI) | P |  | OR (95%CI) | P |  | OR (95%CI) | P |
| PCT |  |  |  |  |  |  |  |  |
| 0 | 1.0 (Reference) |  |  | 1.0 (Reference) |  |  | 1.0 (Reference) |  |
| 1 | 3.891  (1.332, 8.356) | 0.008 |  | 2.959  (0.917, 7.048) | 0.011 |  | 3.528  (1.729, 8.192) | 0.009 |
| 2 | 4.966  (1.718, 9.678) | 0.011 |  | 4.378  (2.122, 9.906) | 0.007 |  | 4.778  (2.165, 9.648) | 0.009 |
| 3 | 7.451  (3.772, 17.658) | 0.002 |  | 6.332  (3.285, 16.968) | 0.001 |  | 6.395  (2.875, 17.235) | 0.001 |
| CRP |  |  |  |  |  |  |  |  |
| 0 | 1.0 (Reference) |  |  | 1.0 (Reference) |  |  | 1.0 (Reference) |  |
| 1 | 1.055  (0.658, 2.177) | 0.279 |  | 0.784  (0.427, 1.682) | 0.587 |  | 0.895  (0.404, 1.782) | 0.445 |
| 2 | 1.195  (0.688, 2.396) | 0.325 |  | 0.856  (0.497, 1.892) | 0.792 |  | 0.967  (0.511, 1.975) | 0.587 |
| dCHC |  |  |  |  |  |  |  |  |
| 0 | 1.0 (Reference) |  |  | 1.0 (Reference) |  |  | 1.0 (Reference) |  |
| 1 | 5.775  (3.593, 15.875) | 0.001 |  | 4.689  (3.152, 9.372) | 0.002 |  | 5.158  (3.336, 12.849) | 0.001 |
| 2 | 7.796  (3.749, 19.395) | 0.006 |  | 7.735  (3.682, 18.483) | 0.001 |  | 7.764  (3.715, 18.768) | 0.003 |
| sNFI |  |  |  |  |  |  |  |  |
| 0 | 1.0 (Reference) |  |  | 1.0 (Reference) |  |  | 1.0 (Reference) |  |
| 1 | 3.681  (2.840, 7.438) | 0.007 |  | 3.591  (1.983, 7.398) | 0.001 |  | 3.609  (2.335, 7.421) | 0.003 |
| 2 | 8.692  (3.881, 22.482) | 0.002 |  | 7.692  (3.752, 18.982) | 0.001 |  | 8.276  (3.754, 20.397) | 0.001 |
| WBC |  |  |  |  |  |  |  |  |
| 0 | 1.0 (Reference) |  |  | 1.0 (Reference) |  |  | 1.0 (Reference) |  |
| 1 | 0.835  (0.466, 1.587) | 0.605 |  | 0.674  (0.398, 1.495) | 0.816 |  | 0.774  (0.396, 1.488) | 0.775 |

SBP: Spontaneous bacterial peritonitis; PCT: procalcitonin; CRP: C-reactive protein; dCHC: difference in hemoglobin concentration between newly formed and mature red blood cells; sNFII: mean fluorescence intensity of mature (segmented) neutrophils; WBC: White blood cell.
